# Intraoperative administration of isoflurane improves survival in rats exposed to caecal ligation and puncture

**DOI:** 10.1016/j.bjao.2022.100014

**Published:** 2022-05-21

**Authors:** Keita Ikeda, Hari P. Osuru, Robert H. Thiele

**Affiliations:** University of Virginia Health System, Department of Anaesthesiology, Charlottesville, VA, USA

**Keywords:** caecal ligature and puncture, isoflurane, propofol, rat, sepsis, survivability, volatile anaesthetics

## Abstract

**Background:**

Emerging data suggest that volatile anaesthetic agents may be protective during critical illness.

**Methods:**

Three-month-old Sprague Dawley rats were randomly allocated to one of four groups: isoflurane during surgery followed by 3 days of isoflurane 0.8% (and intralipid i.v.), propofol during surgery and 314 μg kg^−1^ h^−1^ propofol for 3 days, isoflurane during surgery and intralipid for 3 days, and propofol during surgery and intralipid for 3 days. After induction with propofol or isoflurane, rats breathed oxygen 100% spontaneously via a nose cone. Propofol or intralipid was administered through a 22-gauge jugular vein i.v. catheter. Caecal ligation and puncture was performed through a paramedian incision. The surgical concentration of isoflurane was kept at 2%, and propofol was maintained at 800 μg kg^−1^ h^−1^. After recovery and 3 days of exposure to intralipid or anaesthetic agents, the rats were allowed to roam free in an adequately vented, temperature- and humidity-controlled cage with food and water *ad libitum*.

**Results:**

Rats that received isoflurane for 3 days survived longer than the postoperative propofol group (*P*=0.0002, log-rank test). Among rats receiving no postoperative anaesthetic, those receiving isoflurane during surgery survived longer than those that received propofol during surgery group (*P*=0.0081).

**Conclusions:**

Exposure to isoflurane, as opposed to propofol, may improve survival in rats exposed to caecal ligation and puncture.

The mortality from sepsis is ∼20%^1^ and there have been no sepsis-specific treatment developments for several decades. Volatile anaesthetics are known to provide organ protection after hypoxia or ischaemia–reperfusion events (pharmacological pre-conditioning). Recently, several authors have asked whether or not these same protective properties might manifest during exposure to infection and inflammation. Several studies have suggested that pre-conditioning using volatile anaesthetics may provide organ-specific benefits in animal models of sepsis.^2^, ^3^, ^4^, ^5^, ^6^, ^7^ Studies by Flondor and colleagues,^8^ Boost and colleagues,^9^ and Zhang and colleagues^10^ suggest that the protective pre-conditioning effects of volatile anaesthetics are mediated by modulating the proinflammatory pathways, such as the cytokine response. Thiele and colleagues^11^ and Osuru and colleagues^12^ showed that volatile anaesthetics increased hypoxia-inducible factor 1a expression despite a lack of hypoxia, increased oxidative stress in the brain and the liver, and altered oxygen transport chain activity. Schläpfer and colleagues^13^ discovered that volatile anaesthetics offer a survival benefit compared with propofol in sedated and mechanically ventilated Wistar rats that underwent caecal ligation and puncture (CLP).^13^^,^^14^

In this study we aimed to expand upon Schläpfer's work utilising a longer-term anaesthetic regimen that did not require tracheal intubation and continuous mechanical ventilation to more accurately reflect the conditions experienced by critically ill patients with sepsis and balance the sexes of the groups. We hypothesised that a 72-h course of volatile anaesthetic exposure would lead to a survival benefit compared with a 72-h course of propofol in non-ventilated rats, similar to the results in the 24-h course of volatile anaesthetics and propofol in ventilated rats as demonstrated by Schläpfer and colleagues.^13^

## Methods

### Animals

The University of Virginia Institutional Animal Care and Use Committee (IACUC) approved and registered this study. Pathogen-free, genetically unmodified, 3-month-old Sprague Dawley rats obtained from Envigo (Indianapolis, IN, USA) were housed in standard cages (Allentown, Inc., Allentown, NJ, USA) with food and water *ad libitum* until the time of the experiment. The animals were kept in a University of Virginia vivarium, with a regular 12-h light cycle, food, and water *ad libitum*, and a toy for environmental enrichment for a minimum of 48 h to acclimate to the new surroundings before participating in the study in pairs. The rats supplied by Envigo were examined to be healthy by the vivarium veterinarian after their arrival and observed for an additional 2 days (4 days total) to ensure that they were in good health. All animals had a jugular vein access catheter implanted at the Envigo facility before delivery to our laboratory.

### Experimental procedures

In order to infuse propofol or intralipid without any need for recent exposure to volatile anaesthetics, the animals were purchased from the supplier (Envigo) with a tunnelled external jugular venous catheter already implanted. Induction and maintenance of anaesthesia was with either propofol (i.v. bolus of 2 mg kg^−1^ followed by an infusion at an average of 600 μg kg^−1^ min^−1^, Medfusion 3500 syringe pump, Smiths Medical, Minneapolis, MN) or isoflurane (maintained at 2.0 MAC using an EZ-108SA-NV single animal anaesthesia machine, EZ-178 Sure-Seal induction chamber; E-Z Systems Inc., P.O. Box 3544, Palmer, PA 18043). After confirmation of general anaesthesia, each animal underwent CLP surgery as previously described by our laboratory.^12^ For this particular study, ∼80% of the caecum was ligated and punctured twice with a sterile 16-gauge needle and tied off with size 0 silk sutures. The incision was closed with a double silk 4-0 suture. The temperature and SpO_2_ were monitored using an AD Instruments LabChart 8.1 (Boulder, CO, USA). After the surgical procedure, lidocaine 2% was infiltrated around the incision site and an s.c. injection of 3 ml normal saline was given to compensate for the loss of appetite after surgery. The animals were then allowed to wake up and placed back in their cage. Animals were housed in a hood and exposed to light from 6 AM to 6 PM, dark from 6 PM to 6 AM. All rats were placed under video surveillance once they entered the postoperative part of the study. From the videos, we determined their time of death. Five days after the start of the experiment, animals that survived were euthanised under general anaesthesia.

### Study interventions

**Fig 1:**
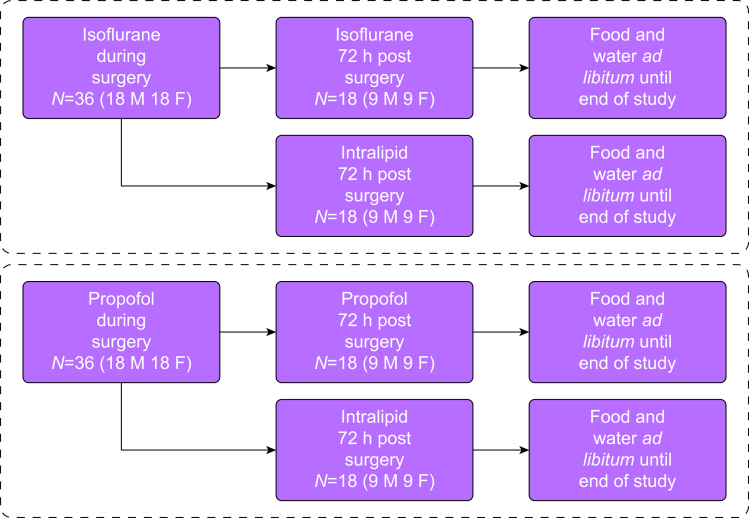
Study design and sample sizes. F, female; M, male.

Rats were randomly allocated to one of four groups; the short propofol group (SP), which received intraoperative propofol and postoperative intralipid for 72 h, the long propofol group (LP), which received intraoperative propofol and postoperative propofol for 72 h, the short isoflurane group (SI), which received intraoperative isoflurane and postoperative intralipid for 72 h, and the long isoflurane group (LI), which received intraoperative isoflurane and postoperative isoflurane for 72 h (Fig. 1).

After the surgical procedure, animals in Groups LP and LI were sedated for 10-h intervals, with 2 h off sedation, to mimic the daily ‘sedation holiday’ strategies used in human ICUs. Sedation was titrated to ∼80% of a general anaesthetic dose (propofol infusion rate 317 μg kg^−1^ min^−1^ or isoflurane 0.7 MAC), thus avoiding the need for tracheal intubation and mechanical ventilation of the lungs: all sedated animals were kept in an oxygen 100% environment during their 3-day postsurgical anaesthetic regimen. Because propofol infusions result in the administration of fluids and calories (fat), animals not receiving propofol (which contains 1.1 kcal ml^−1^) received intralipid 10% (1.0 kcal ml^−1^), as described by Schläpfer and colleagues^13^ We infused intralipid 10% at 349 μg kg^−1^ min^−1^, for equivalent administration of fluid and calories (assuming a propofol infusion rate of 317 μg kg^−1^ min^−1^ [5.87 ml h^−1^]). All infusions were controlled using a Medfusion 3500 syringe pump.

#### Outcomes

The primary endpoint of this study was survival after CLP, with animals euthanised at an endpoint defined by IACUC's ethical endpoint policy, which was derived from pain studies by Sotocinal and colleagues^15^ and Demartini and colleagues.^16^ We applied criteria for the euthanasia scoring system to each animal daily after the procedure (Table 1). The variables in Table 1 were assessed every 8–12 h in mice subjected to CLP. A combined score of 8 or maximal scores in two or more areas (e.g. activity and weight) will trigger euthanasia.

**Table 1:** Euthanasia scoring scheme.

Body weight	
0	Normal
1	<10%
2	10–19%
3	≥20%
Physical condition	
Haircoat	
0	Normal
1	Rough haircoat
2	Rough coat, hair loss, ungroomed
Eyes and nose	
0	Normal
1	Eyes close or squinted (no discharge)
2	Eyes close or squinted (discharge or porphyrin staining)
Behaviour	
Activity	
0	Normal
1	Decreased activity, locomotion after slight stimulation
2	Inactive, less alert, locomotion after moderate stimulation
3	Self-mutilation, very restless or immobile or no locomotion after moderate stimulation
Posture	
0	Normal
1	Sitting in hunched up position
2	Hunched posture/head on cage floor
3	Lying prone on cage floor
Additional criteria for euthanasia (even if the total score is <8) include:	
1	Weight loss > 20% which cannot be corrected in 2 days by dietary supplementation
2	Two consecutive rectal or infrared temperatures are <32°C

### Randomisation and blinding

We randomised the selection of rats into the four groups by numbering the recently arrived rats and randomly assigning them to the four groups, according to their sex. The males and females were kept separately, and they were randomised independently. Only the animal surgeon was aware of the group allocation.

### Statistical methods

#### Sample size

We based our sample size on a previous study examining sepsis outcome in rats by Schläpfer and colleagues,^13^ which used approximately nine animals per group. We considered an increase in survival of 12 h to be a biologically relevant change and a reasonable estimate of variability to be about 10 h. We set the risk of obtaining a false positive to be 1 in 20 for a single statistical test or a significance threshold of alpha=0.05. Rats were divided into eight subgroups of approximately nine rats per subgroup—36 rats were exposed to isoflurane and 36 to propofol. In each anaesthetic agent group, rats were further divided into an extended anaesthesia subgroup and a no postoperative anaesthesia subgroup (18 rats in each subgroup). In each subgroup there were an equal number of females (nine) and males (nine), as in Figure 1. Protocol deviation, such as incorrect administration of anaesthetic agents and equipment failure, resulted in exclusion of the animal from the analysis and its replacement.

### Analyses

Survival after CLP was determined using Kaplan–Meier survival curves. Comparisons between curves were made using the log-rank test, which uses the Mantel–Haenszel method^17^ to handle multiple deaths in the same time period to test the null hypothesis that one treatment was better than the other. The log-rank test shares the same assumptions as of the Kaplan–Meier survival curve and the Mantel–Haenszel method and is equally weighted throughout the experiment. As we had no censoring, the survival probabilities are only affected by the difference in the groups being compared. We also compared the slopes of the Kaplan–Meier curves to determine the ratio of the rates of death, or the hazard ratio,^18^ from the Mantel–Haenszel method, to see if one method was associated with significantly higher mortality than the other. All statistical calculations were performed with GraphPad Prism 9.1 (GraphPad, La Jolla, CA, USA).

## Results

**Fig 2:**
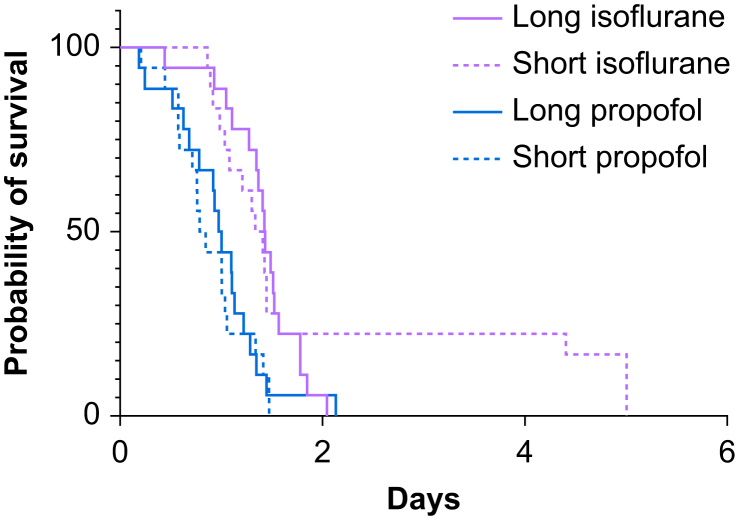
Kaplan–Meyer survival data comparisons for all groups.

**Fig 3:**
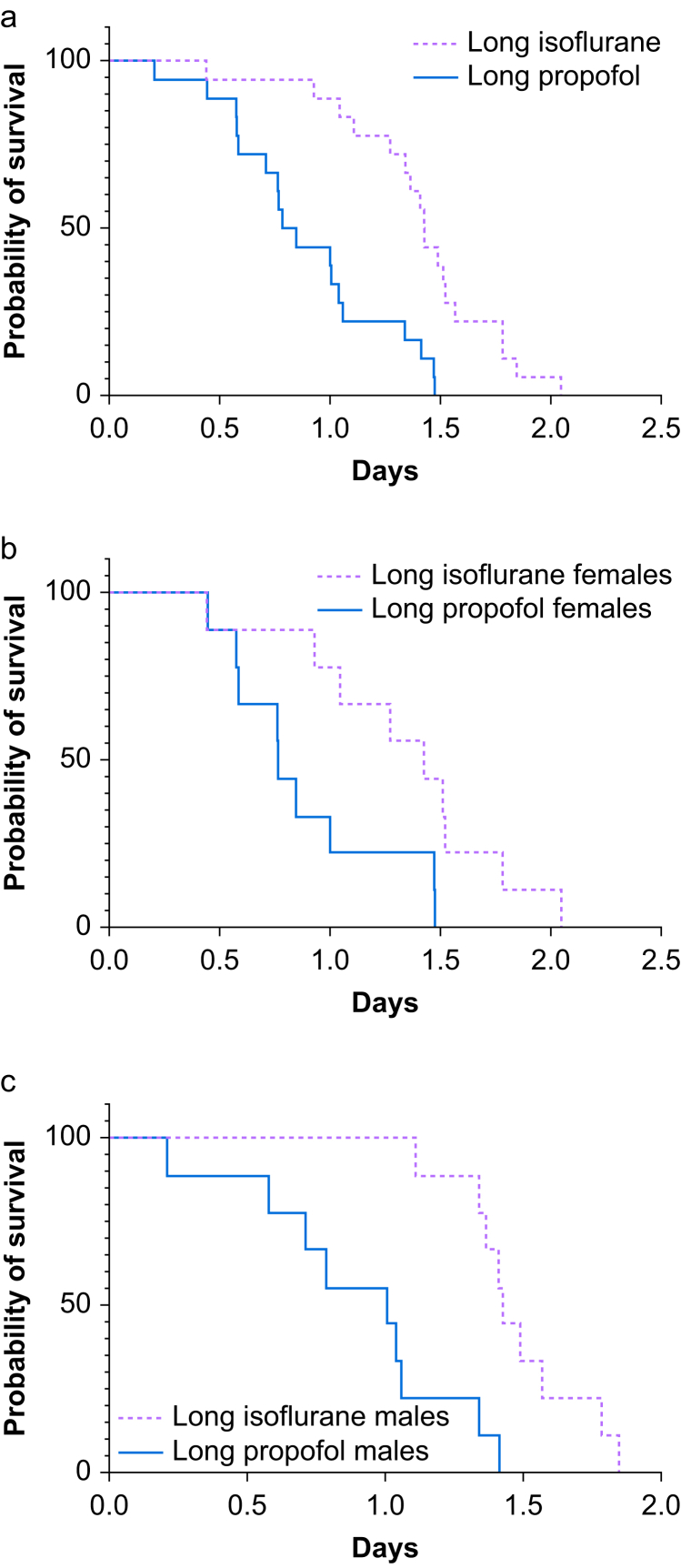
Kaplan–Meyer survival data comparisons for groups LI and LP. (a) For males and females together, *P*<0.0001. (b) For groups LI and LP females, *P*=0.0224. (c) For groups LI and LP males, *P*=0.0007. LI, long isoflurane group; LP, long propofol group.

**Fig 4:**
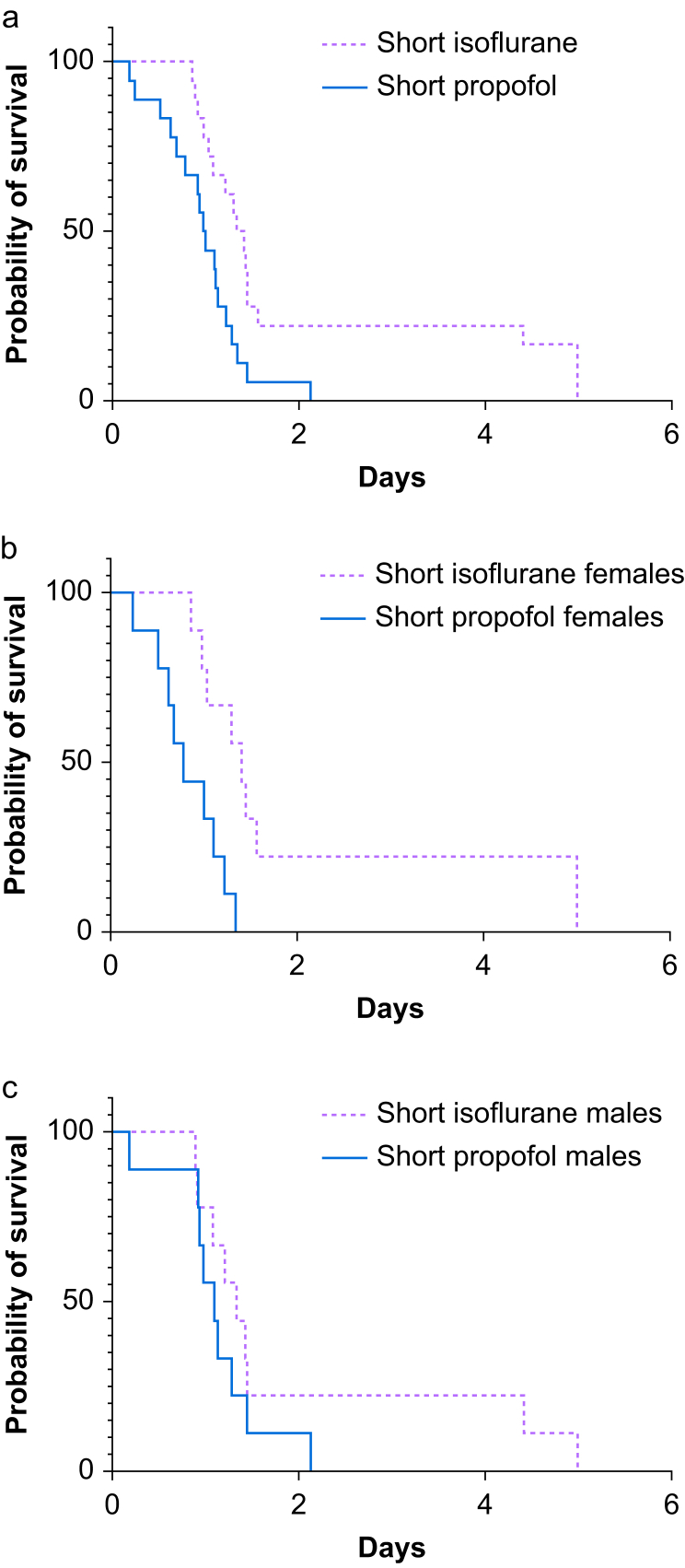
Kaplan–Meyer survival data comparisons for groups SI and SP. (a) For males and females together, *P*<0.0001. (b) For groups SI and SP females, *P*=0.0224. (c) For groups SI and SP males, *P*=0.0007. SI, short isoflurane group; SP, short propofol group.

A total of 72 animals underwent CLP. The weights of the rats, according to the group and sex, are shown in Supplementary Table S3. Survival data are presented in Fig 2, Fig 3, Fig 4. Survival comparisons of all groups are shown in Figure 2.

### Comparison of long isoflurane *vs* long propofol sedation on survival

The log-rank test for postoperative anaesthetic agent groups showed that rats in group LI survived longer than those in group LP (*P*<0.0001, Fig. 3, Supplementary Table S4), and the median survival times were 1.44 and 0.82 days, respectively. The ratio of the medians showed that rats in group LP were 1.75 times more likely to die than those in group LI. The hazard ratio calculated with the Mantel–Haenszel method showed rats in group LP to be 5.27 times more likely to die than those in group LI, and the hazard ratio calculated with the log-rank method was 3.13. A separate analysis of each sex of mice showed a statistically significant difference in females (*P*=0.0224) and males (*P*=0.0005) in groups LI and LP.

### Comparison of short isoflurane *vs* short propofol sedation on survival

The log-rank test comparison between groups SI and SP showed that rats in group SI that received isoflurane anaesthesia for surgery and intralipid postoperatively survived longer than rats in group SP (*P*=0.006, Fig. 4, Supplementary Table S5), and the median survival times were 1.37 and 0.99 for groups SI and SP, respectively. The hazard ratio calculated with the Mantel–Haenszel method and the log-rank methods were 2.86 and 2.33, respectively. A separate analysis of the sexes showed a significant difference between group SI and SP females (*P*=0.0053) but no statistically significant difference in males.

### Comparison of long isoflurane *vs* short isoflurane sedation on survival

There was no statistically significant difference in survival between groups LI and SI, either when the sexes were analysed together or separately.

### Comparison of long propofol *vs* short propofol sedation on survival

There was no statistically significant difference in survival between groups LP and SP, either when the sexes were analysed together or separately.

## Discussion

We found that rats exposed to intraoperative and postoperative isoflurane lived longer than rats exposed to intraoperative and postoperative propofol after CLP. Similarly, we found that rats exposed to intraoperative isoflurane (and no postoperative sedation) lived longer than rats exposed to intraoperative propofol (and no postoperative sedation) after CLP. The dose of isoflurane we could deliver postoperatively was limited by our decision not to intubate and mechanically intubate these animals. That said, a comparison of rats exposed to isoflurane both intraoperatively and postoperatively, compared with those exposed to only intraoperative isoflurane, suggests that in our protocol, the intraoperative isoflurane was most beneficial. This was a surprise and suggested that a brief course of isoflurane is protective. We did not test a group that was exposed to intraoperative propofol and postoperative isoflurane, but such a study is planned. This would allow us to determine the optimal timing of isoflurane exposure and has practical implications.

The time course of isoflurane exposure matters because the primary infection in 39% of sepsis cases is the lungs.^19^ In these patients, many of whom have community-acquired pneumonia, isoflurane exposure can only occur after the initial infection. Still, 30% of septic cases occur in surgical patients^20^ and volatile anaesthetic exposure during the surgical procedure may help improve outcomes in these patients.

A secondary finding is that our data suggest that there may be sex differences in the survival benefit secondary to isoflurane anaesthesia compared with propofol anaesthesia. However, when isoflurane sedation was used after surgery, the survival benefit compared with postsurgical propofol sedation was present in males and females. Sex- and gender-based differences in response to sepsis and inflammation have been reported previously.^21^, ^22^, ^23^

Our study has several limitations. First, it is an animal study, and the results are not directly applicable to humans. Second, CLP does not perfectly mimic human sepsis. This potential discrepancy can be somewhat mitigated by adhering to the Minimum Quality Threshold in Pre-clinical Sepsis Studies (MQTiPSS) guidelines, which were not followed in this manuscript (in part because the 72 h postoperative nature of this study made monitoring and haemodynamic management impractical). Third, we did not quantify the impact of CLP on inflammation or measure sepsis severity. Fourth, as described above, we did not test propofol-isoflurane or isoflurane-propofol regimens (to determine if postoperative addition of isoflurane would be protective), but such a study is planned. Fifth, the dose of isoflurane and propofol we could deliver postoperatively was limited by our inability to continuously monitor *and* intervene on behalf of our animals, which would not be the case in an ICU.

## Conclusions

Exposure of rats to isoflurane may prolong survival after CLP as compared with exposure to propofol. This was true for rats with limited exposure during surgery with no postoperative anaesthetics and those receiving intraoperative isoflurane with postoperative isoflurane sedation.

## Authors' contributions

Data acquisition: KI, HPO.

Study conception and design, obtained IACUC approval: RHT.

Critically revised drafts of the manuscript, approved the final version, and agree to be accountable for all aspects of the work: all authors.

Keita Ikeda was responsible for analysis and Interpretation of the data and drafting the manuscript.

Robert Thiele was responsible for interpretation of the data and drafting the manuscript.

## Acknowledgements

We would like to thank all the veterinary technicians in the vivarium for helping us with many of the animal husbandry questions and issues. We would also like to acknowledge the staff of the 10.13039/100008457University of Virginia Department of Anesthesiology for their work in supporting us.

## Declarations of interest

The authors declare that they have no conflicts of interest.

## Data availability

The datasets used, analysed, or both during the current study are available from the corresponding author on reasonable request.

## Funding

K-08 grant, 10.13039/100000057National Institute of General Medical Sciences (5K08GM115861-04) to RHT.
