# Supplementary material for: Intraoperative administration of isoflurane improves survival in rats exposed to caecal ligation and puncture
Source: BJA Open. 2022 May 21;2:100014. doi: 10.1016/j.bjao.2022.100014 (PMC10430831; doi:10.1016/j.bjao.2022.100014)
Supplement: Multimedia component 1 [file mmc1.docx]

KEY WORDS:

Volatile Anesthetics, Anesthetic Pharmacology, Sepsis, propofol, Critical Care

| **ARRIVE Guideline 2.0** | | |
| --- | --- | --- |
| **ID** | **Question** | **Answer** |
| 1 | **Study Design – For each experiment, provide brief details of study design** | |
| 1a | The groups being compared, including control groups. If no control group has been used, the rationale should be stated. | This paper addresses a pair of randomized controlled one-way trial with 36 Sprague Dawley rats per trial, evenly weighted for sex, for a total of 72 Sprague Dawley rats. We investigated the effect of long term exposure to anesthetic agents on the survivability to sepsis. First one-way trail was conducted with isoflurane, and the second with Propofol. |
| 1b | The experimental unit (e.g., a single animal, litter, or cage of animals). | Rat |
| 2 | **Sample Size** | |
| 2a | Specify the exact number of experimental units allocated to each group, and the total number in each experiment. Also indicate the total number of animals used. | The rats were divided into two groups – 36 for the Isoflurane group and 36 for the Propoful group. In each anesthetic agent group, they were further divided into the extended anesthesia subgroup and a control subgroup, 18 rats each. In those subgroups, there were equal number of females (9) and males (9).     \| Anesthetic Agent during Surgery \| Post operative Treatment \| N = \| \| --- \| --- \| --- \| \| Isoflurane \| Isoflurane for 72 hours \| 9 Male 9 Female \| \| Intralipid for 72 hours \| 9 Male 9 Female \| \| Propofol \| Propofol for 72 hours \| 9 Male 9 Female \| \| Intralipid for 72 hours \| 9 Male 9 Female \| |
| 2b | Explain how the sample size was decided. Provide details of any a priori sample size calculation, if done. | We based the required sample size based on a previous study examining sepsis outcome in rats by Schlapfer et al [1].  [1] Schläpfer M, Piegeler T, Dull RO, Schwartz DE, Mao M, Bonini MG, Z'Graggen BR, Beck-Schimmer B, Minshall RD. Propofol increases morbidity and mortality in a rat model of sepsis. Crit Care. 2015 Feb 19;19(1):45. doi: 10.1186/s13054-015-0751-x. PMID: 25887642; PMCID: PMC4344774.  **The primary objective of the experiment – what is the main outcome measure?**  The primary outcome measure is the Kaplan-Meier survivability analysis and we tested all the possible combination of hypothesis regarding enhanced survivability using the log-rang (LR) test.  **The predefined effect size – What is a biologically relevant effect size?**  We considered a survivability increase of 12 hours to be a biologically relevant change in survivability.  **What is the estimate of variability?**  According to Schlapfer et al, a reasonable estimate of variability is about 10 hours.  **Significance threshold – what risk of a false positive is acceptable?**  We set the risk of obtaining a false positive to be 1 in 20 for a single statistical test, or a significance threshold of alpha = 0.05.  **Significance threshold—What risk of a false positive is acceptable?**  The significance level (alpha) is set at 0.05 and the risk of obtaining a false positive is 1 in 20.  **Power—What risk of a false negative is acceptable?**  The power (1 – beta) is 5%.  **Directionality—Will you use a one- or two-sided test?**  This is a single sided test. |
| 3 | **Inclusion and exclusion criteria** | |
| 3a | Describe any criteria used for including or excluding animals (or experimental units) during the experiment, and data points during the analysis. Specify if these criteria were established a priori. If no criteria were set, state this explicitly. | Rats that experienced protocol deviation, such as improper administration of anesthetic agents and equipment failure, were excluded from the analysis and were replaced. |
| 3b | For each experimental group, report any animals, experimental units, or data points not included in the analysis and explain why. If there were no exclusions, state so. | Three rats were excluded and replaced during the experiment. One female rat in the isoflurane group did not receive intralipid post op when the syringe pump failed, another rat developed an occlusion in the IV access, and another rat experienced high temperature due to air conditioning malfunction. |
| 3c | For each analysis, report the exact value of n in each experimental group. | The three rats which experienced protocol deviations were replaced to maintain a sample number of 9 per group. |
| 4 | **Randomization** | |
| 4a | State whether randomization was used to allocate experimental units to control and treatment groups. If done, provide the method used to generate the randomization sequence. | All animals were simultaneously randomized to the treatment groups, and the randomization was constrained by the number of animals that could be ordered at a time, and how many animals could undergo the experiment per week. |
| 4b | Describe the strategy used to minimize potential confounders such as the order of treatments and measurements, or animal/cage location. If confounders were not controlled, state this explicitly. | Three month old Sprague-Dawley rats were obtained from Envigo (Madison, WI, USA) in multiples of four. Because males and females were kept separately, they were randomized separately. |
| 5 | **Blinding** | |
|  | Describe who was aware of the group allocation at the different stages of the experiment (during the allocation, the conduct of the experiment, the outcome assessment, and the data analysis). | Only the animal surgeon was aware of the group allocation for each stage of the experiment. |
| 6 | **Outcome measures** | |
| 6a | Clearly define all outcome measures assessed (e.g., cell death, molecular markers, or behavioral changes). | We measured the hours of survival for the different treatment and their controls. |
| 6b | For hypothesis-testing studies, specify the primary outcome measure, i.e., the outcome measure that was used to determine the sample size. | The primary endpoint of this study was defined as the ethical endpoint as per IACUC policy |
| 7 | **Statistical methods** | |
| 7a | Provide details of the statistical methods used for each analysis, including software used. | The Kaplan-Meier survival curves and the log-rang test to test the null hypothesis were done were performed with GraphPad Prism 9.1 (GraphPad, La Jolla, CA, USA). Additional statistical analysis was performed with Microsoft Excel (Redmond, WA, USA). Three animals that experienced protocol deviation due to equipment malfunction were excluded and replaced. |
| 7b | Describe any methods used to assess whether the data met the assumptions of the statistical approach, and what was done if the assumptions were not met. | The logrank test shares the same assumptions as the Kaplan-Meier survival curve. Since we have had no censoring, the survival probabilities are only affected by the difference in the groups being compared. |
| 8 | **Experimental Animals** | |
| 8a | Provide species-appropriate details of the animals used, including species, strain and substrain, sex, age or developmental stage, and, if relevant, weight. | Because sepsis leads to multi-organ dysfunction and death, both of which we are studying, it is not possible to replace animal studies with non- animal techniques. We excluded murine and baboon models due to their resilience to toxic effects of liposaccharides, whereas humans are not [1]. Therefore, we settled on the rodent model, specifically the Sprague Dawley for their docile nature and their growth potential. Also, Sprague Dawley rats tend to be more suitable for pharmacokinetic alterations [2]. We chose three month old rats as they are just beyond the sexual maturity [3]. We also wanted to extend the work done by Schläpfer to include female rats of similar age.  [1] Fink MP. Animal models of sepsis. Virulence. 2014 Jan 1;5(1):143-53. doi: 10.4161/viru.26083. Epub 2013 Aug 19. PMID: 24022070; PMCID: PMC3916368.  <https://www.ncbi.nlm.nih.gov/pmc/articles/PMC3916368/>  [2] García-López P, Pérez-Urizar J, Ibarra A, Grijalva I, Madrazo I, Flores-Murrieta F, Castañeda-Hernández G, Guízar-Sahagún G. Comparison between Sprague-Dawley and Wistar rats as an experimental model of pharmacokinetic alterations induced by spinal cord injury. Arch Med Res. 1996 Winter;27(4):453-7. PMID: 8987177.  [3] Sengupta P. The Laboratory Rat: Relating Its Age With Human's. Int J Prev Med. 2013 Jun;4(6):624-30. PMID: 23930179; PMCID: PMC3733029. |
| 8b | Provide further relevant information on the provenance of animals, health/immune status, genetic modification status, genotype, and any previous procedures. | The rats supplied by Envigo were not genetically modified and were examined to be healthy by the vivarium veterinarian after their arrival. They were observed for two additional days and were found to be in good health. The Propofol group of animals had jugular vein access catheter implanted at Envigo facility before delivery to our lab. |
| 9 | **Experimental Procedures** | |
| 9a | What was done, how it was done, and what was used. | Each animal underwent a cecal ligation and puncture (CLP) surgery where approximately 80% of the cecum was ligated and punctured twice with a sterile 16 gauge needle. The anesthetic protocol for surgery was divided into two groups, and those two groups were subdivided for their post-surgical anesthetic protocol for a total of four groups.  In order to infuse Propofol, the Propofol group of animals were purchased from the supplier (Envigo) with a tunneled external jugular venous catheter already implanted – which was more reliable than a tail vein. Additionally, because Propofol infusions result in administration of fluids and calories (fat), animals not-receiving Propofol (1.1 kcal/mL) will receive an equipotent dose of 10% Intralipid (1.0 kcal/mL), as described by Schlapfer et al. Crit Care 19: 45, 2015. We anticipate that the Propofol infusion rate will be 317 ucg/kg/min (5.87 mL/hr) and that the 10% Intralipid infusion rate will be 349 ucg/kg/min, for equivalent administration of fluid and calories. We chose 10% Intralipid instead of 11% for ease of preparation and increased the flow rate by 10% for equipotent dose.  Group 1: Surgical sedation of isoflurane anesthesia (2.0 MAC), and post-surgical sedation with isoflurane (approximately 0.7 MAC) for 3 days. After the three days, they are allowed to eat and drink ad libitum.  Group 2: Surgical sedation of isoflurane anesthesia (2.0 MAC), no post-surgical sedation, Intralipid for 3 days. After the three days, they are allowed to eat and drink ad libitum. This group serves as the control for extended isoflurane exposure of group 1.  Group 3: Surgical sedation of Propofol anesthesia (avg. 600 ucg/kg/min), and post-surgical sedation with Propofol (317 ucg/kg/min) for 3 days. After the three days, they are allowed to eat and drink ad libitum. Of note, these animals will be received from Envigo with venous catheters already implanted.  Group 4: Surgical sedation of Propofol anesthesia (avg. 600 ucg/kg/min), no post-surgical sedation, and Intralipid for 3 days. After the three days, they are allowed to eat and drink ad libitum. Of note, these animals will be received from Envigo with venous catheters already implanted. This group serves as the control for extended Propofol exposure of group 3.  Groups 1 and 2 were induced in an induction chamber and immediately transferred to a nose cone after induction. Groups 3 and 4 were induced with a Propofol injection of 2000 ug/kg/min until loss of consciousness, then transferred to a nose cone, and the surgical infusion rate of Propofol was adjusted down on average to 600 ug/kg/min.  They were allowed to wake up and placed back in their cage. They then were sedated for 10 hour intervals, with 2 hours off sedation, to mimic the daily sedation holiday strategies employed in human intensive care units. Sedation was titrated to approximately 80% of a general anesthetic dose, therefore did not require intubation or ventilation, and received supplemental oxygenation in their cages. Animals were housed in a hood and exposed to light from 6A-6P, dark from 6P-6A. After 5 days from start of experiment, animals were euthanized under general anesthesia.  All animals were under constant surveillance and monitored for signs of pain and distress twice a day for the first three days, then once a day for day 4 and day 5, and given Nocita (bupivacaine sustained release) 5.3 mg/kg (0.4 mL/kg) Q72h injected around the incision site. We used a scoring system that was developed by Dr. Shawn Rosensteel from ACUC at the University of Virginia to score each animal daily after the procedure. |
| 9b | When and how often. | N/A |
| 9c | Where (including detail of any acclimatisation periods). | The animals were allowed to acclimate to the University of Virginia vivarium for two days before research activities on the third day. |
| 9d | Why (provide rationale for procedures). |  |
| 10 | **Results** | |
| 10a | Summary/descriptive statistics for each experimental group, with a measure of variability where applicable (e.g., mean and SD, or median and range). | \| Group \| Gender \|  \| Mass before CLP \| Mass at death (g) \| Change in Mass \| \| --- \| --- \| --- \| --- \| --- \| --- \| \| Isoflurane surgery \| F \| Mean \| 267.33 \| 265.56 \| -1.78 \| \| Isoflurane Post Op \|  \| Median \| 266.00 \| 262.00 \| -2.00 \| \|  \|  \| SD \| 22.19 \| 21.76 \| 1.93 \| \| Isoflurane surgery \| F \| Mean \| 263.33 \| 252.33 \| -11.00 \| \| Intralipid Post Op \|  \| Median \| 261.00 \| 250.00 \| -5.00 \| \|  \|  \| SD \| 14.02 \| 15.08 \| 10.17 \| \| Propofol Surgery \| F \| Mean \| 265.22 \| 261.78 \| -3.44 \| \| Intralipid Post Op \|  \| Median \| 265.00 \| 257.00 \| -3.00 \| \|  \|  \| SD \| 14.32 \| 13.98 \| 2.17 \| \| Propofol Surgery \| F \| Mean \| 261.89 \| 256.67 \| -5.22 \| \| Propofol Post Op \|  \| Median \| 256.00 \| 250.00 \| -2.00 \| \|  \|  \| SD \| 13.63 \| 13.89 \| 9.51 \| \| Isoflurane surgery \| M \| Mean \| 307.44 \| 305.44 \| -2.00 \| \| Isoflurane Post Op \|  \| Median \| 294.00 \| 290.00 \| 0.00 \| \|  \|  \| SD \| 27.02 \| 28.30 \| 3.89 \| \| Isoflurane surgery \| M \| Mean \| 299.78 \| 287.22 \| -12.56 \| \| Intralipid Post Op \|  \| Median \| 307.00 \| 290.00 \| -6.00 \| \|  \|  \| SD \| 15.54 \| 19.43 \| 15.79 \| \| Propofol Surgery \| M \| Mean \| 298.33 \| 294.56 \| -3.78 \| \| Intralipid Post Op \|  \| Median \| 298.00 \| 281.00 \| -3.00 \| \|  \|  \| SD \| 16.31 \| 20.73 \| 7.63 \| \| Propofol Surgery \| M \| Mean \| 309.11 \| 308.44 \| -0.67 \| \| Propofol Post Op \|  \| Median \| 296.00 \| 296.00 \| 0.00 \| \|  \|  \| SD \| 31.57 \| 34.11 \| 4.42 \|   LR test for post op anesthetic agent groups showed rats that received isoflurane for three days survived longer than the propofol group (P = 0.0002, Figure 1). Within the post op no anesthetic agent (control) group, the group that received isoflurane during surgery survived longer than the propofol group (P = 0.0081, Figure 2). Within the control group, the male rats did not perform well with propofol against isoflurane (P = 0.2599).       \| Comparison of Survival Curves \|  \|  \| \| --- \| --- \| --- \| \|  \|  \|  \| \| Log-rank (Mantel-Cox) test \|  \|  \| \| Chi square \| 15.44 \|  \| \| df \| 1 \|  \| \| P value \| <0.0001 \|  \| \| P value summary \| **** \|  \| \| Are the survival curves sig different? \| Yes \|  \| \|  \|  \|  \| \| Gehan-Breslow-Wilcoxon test \|  \|  \| \| Chi square \| 13.64 \|  \| \| df \| 1 \|  \| \| P value \| 0.0002 \|  \| \| P value summary \| *** \|  \| \| Are the survival curves sig different? \| Yes \|  \| \|  \|  \|  \| \| Median survival \|  \|  \| \| Isoflurane During Surgery, Isoflurane Post Op 72 Hours \| 1.425 \|  \| \| Propofol During Surgery, Propofol Post Op 72 Hours \| 0.8163 \|  \| \| Ratio (and its reciprocal) \| 1.746 \| 0.5729 \| \| 95% CI of ratio \| [0.9083, 3.355] \| [0.2981, 1.101] \| \|  \|  \|  \| \| Hazard Ratio (Mantel-Haenszel) \| A/B \| B/A \| \| Ratio (and its reciprocal) \| 0.1898 \| 5.269 \| \| 95% CI of ratio \| [0.08284, 0.4348] \| [2.300, 12.07] \| \|  \|  \|  \| \| Hazard Ratio (logrank) \| A/B \| B/A \| \| Ratio (and its reciprocal) \| 0.3192 \| 3.133 \| \| 95% CI of ratio \| [0.1489, 0.6844] \| [1.461, 6.717] \|  \| Comparison of Survival Curves \|  \|  \| \| --- \| --- \| --- \| \|  \|  \|  \| \| Log-rank (Mantel-Cox) test \|  \|  \| \| Chi square \| 7.553 \|  \| \| df \| 1 \|  \| \| P value \| 0.006 \|  \| \| P value summary \| ** \|  \| \| Are the survival curves sig different? \| Yes \|  \| \|  \|  \|  \| \| Gehan-Breslow-Wilcoxon test \|  \|  \| \| Chi square \| 7.015 \|  \| \| df \| 1 \|  \| \| P value \| 0.0081 \|  \| \| P value summary \| ** \|  \| \| Are the survival curves sig different? \| Yes \|  \| \|  \|  \|  \| \| Median survival \|  \|  \| \| Isoflurane During Surgery, Intralipid Post Op 72 Hours \| 1.372 \|  \| \| Propofol During Surgery, Intralipid Post Op 72 Hours \| 0.9872 \|  \| \| Ratio (and its reciprocal) \| 1.389 \| 0.7197 \| \| 95% CI of ratio \| [0.7229, 2.670] \| [0.3745, 1.383] \| \|  \|  \|  \| \| Hazard Ratio (Mantel-Haenszel) \| A/B \| B/A \| \| Ratio (and its reciprocal) \| 0.3492 \| 2.864 \| \| 95% CI of ratio \| [0.1649, 0.7395] \| [1.352, 6.066] \| \|  \|  \|  \| \| Hazard Ratio (logrank) \| A/B \| B/A \| \| Ratio (and its reciprocal) \| 0.4298 \| 2.327 \| \| 95% CI of ratio \| [0.2108, 0.8764] \| [1.141, 4.744] \|  \| Comparison of Survival Curves \|  \|  \| \| --- \| --- \| --- \| \|  \|  \|  \| \| Log-rank (Mantel-Cox) test \|  \|  \| \| Chi square \| 0.218 \|  \| \| df \| 1 \|  \| \| P value \| 0.6406 \|  \| \| P value summary \| ns \|  \| \| Are the survival curves sig different? \| No \|  \| \|  \|  \|  \| \| Gehan-Breslow-Wilcoxon test \|  \|  \| \| Chi square \| 0.2555 \|  \| \| df \| 1 \|  \| \| P value \| 0.6133 \|  \| \| P value summary \| ns \|  \| \| Are the survival curves sig different? \| No \|  \| \|  \|  \|  \| \| Median survival \|  \|  \| \| Isoflurane During Surgery, Isoflurane Post Op 72 Hours \| 1.425 \|  \| \| Isoflurane During Surgery, Intralipid Post Op 72 Hours \| 1.372 \|  \| \| Ratio (and its reciprocal) \| 1.039 \| 0.9625 \| \| 95% CI of ratio \| [0.5406, 1.997] \| [0.5008, 1.850] \| \|  \|  \|  \| \| Hazard Ratio (Mantel-Haenszel) \| A/B \| B/A \| \| Ratio (and its reciprocal) \| 1.182 \| 0.8457 \| \| 95% CI of ratio \| [0.5852, 2.389] \| [0.4186, 1.709] \| \|  \|  \|  \| \| Hazard Ratio (logrank) \| A/B \| B/A \| \| Ratio (and its reciprocal) \| 1.156 \| 0.8652 \| \| 95% CI of ratio \| [0.6004, 2.225] \| [0.4494, 1.666] \|  \| Comparison of Survival Curves \|  \|  \| \| --- \| --- \| --- \| \|  \|  \|  \| \| Log-rank (Mantel-Cox) test \|  \|  \| \| Chi square \| 0.2302 \|  \| \| df \| 1 \|  \| \| P value \| 0.6314 \|  \| \| P value summary \| ns \|  \| \| Are the survival curves sig different? \| No \|  \| \|  \|  \|  \| \| Gehan-Breslow-Wilcoxon test \|  \|  \| \| Chi square \| 0.3404 \|  \| \| df \| 1 \|  \| \| P value \| 0.5596 \|  \| \| P value summary \| ns \|  \| \| Are the survival curves sig different? \| No \|  \| \|  \|  \|  \| \| Median survival \|  \|  \| \| Propofol During Surgery, Intralipid Post Op 72 Hours \| 0.9872 \|  \| \| Propofol During Surgery, Propofol Post Op 72 Hours \| 0.8163 \|  \| \| Ratio (and its reciprocal) \| 1.209 \| 0.8269 \| \| 95% CI of ratio \| [0.6292, 2.324] \| [0.4303, 1.589] \| \|  \|  \|  \| \| Hazard Ratio (Mantel-Haenszel) \| A/B \| B/A \| \| Ratio (and its reciprocal) \| 0.8488 \| 1.178 \| \| 95% CI of ratio \| [0.4344, 1.659] \| [0.6029, 2.302] \| \|  \|  \|  \| \| Hazard Ratio (logrank) \| A/B \| B/A \| \| Ratio (and its reciprocal) \| 0.8553 \| 1.169 \| \| 95% CI of ratio \| [0.4441, 1.647] \| [0.6071, 2.252] \| |
| 10b | If applicable, the effect size with a confidence interval. | Effect size is not meaningful in a Kaplan Meier survival analysis – log-rank measures the difference of two survival curves at all time-points in a Kaplan-Meier survival plot. |
| 11 | **Abstract** | |
|  | Provide an accurate summary of the research objectives, animal species, strain and sex, key methods, principal findings, and study conclusions. | TITLE:  Dual one-way study of sepsis survivability of Rats with cecal ligation and puncture due to prolonged exposure to Propofol or Isoflurane  BACKGROUND  Several studies have also shown that volatile anesthetic agent (VAA) affects the pro-inflammatory pathways such as the cytokine response and provide a protective effect that improves the outcome of sepsis. In contrast, Propofol has been shown having no protective effect on sepsis in murine and rat models. The aim of this study was to investigate the efficacy of extended exposure to anesthetics, isoflurane and Propofol.  METHODS  All surgeries were done with a nose cone with 100% oxygen and spontaneous breathing. A 22 gauge intravenous catheter was introduced into the jugular vein for administering Propofol and Intralipid. Sepsis was induced in the rats by performing cecal ligation and puncture (CLP) through a paramedian incision into the abdominal cavity. A total of 72 Sprague Dawley rats, 36 male and 36 female, were randomized into four groups – Isoflurane during surgery followed by three days of 0.8% isoflurane, Propofol during surgery and 314 ug/kg/hr Propofol for three days, isoflurane during surgery and Intralipid for three days, and Propofol during surgery and Intralipid for three days. After the three days, the rats were allowed to roam free in a properly vented, temperature and humidity controlled cage with food and water ad libitum. The surgical concentration of isoflurane was kept at 2%, Propofol was maintained at 800 ug/kg/hr maintenance. Survival data was summarized using Kaplan-Meier curve with Log-Rank (LR) test to determine significance.  RESULTS  Log-Rank test for post op anesthetic agent groups showed that rats that received isoflurane for three days survived longer than the Propofol group (P = 0.0002). Within the post op no anesthetic agent (control) group, the group that received isoflurane during surgery survived longer than the Propofol group (P = 0.0081). Within the control group, the male rats did not perform well with Propofol against isoflurane (P = 0.2599).  CONCLUSIONS:  There were significant difference in survivability of rats exposured to Propofol or isoflurane, for both control and post op anesthetics group. The controls group lived longer on average and rats exposed to isoflurane during the 30-minute CLP surgery improved survivability, which supports the findings of Hermann et al. Also, increased hypoxia-inducible factor-1a expression, despite a lack of hypoxia, increased oxidative stress in the brain, and increased serum lactate (unlike Propofol). These differences between VAA and Propofol may indicate that the inflammatory response induced by the VAA, in the absence of infection, is protective as it prepares the body for a future infection. |
| 12 | **Background** | |
| 12a | Include sufficient scientific background to understand the rationale and context for the study, and explain the experimental approach. | Several studies have been conducted on the protective effects of volatile anesthetic agent (VAA) preconditioning improving the outcome of sepsis ^1,2,3,4,5,6^ . Several studies have also shown that VAA affects the proinflammatory pathways such as the cytokine response ^7,8,9^. In contrast, Propofol has been shown having no protective effect on sepsis in murine and rat models ^10,11^. The aim of this study was to investigate the efficacy of extended exposure to anesthetics, isoflurane and propofol.  Rudd, Kissoon, et al, reported that in 2017, approximately 20% mortality of sepsis cases, and sepsis ranks among one of the leading global burden of disease in poorer nations [12] and meta-analysis of sepsis cases of one healthcare system that covers approximately 20% of United States indicated a 12.5% mortality. [13] In the absence of an effective therapeutic approach for sepsis, Schläpfer et al demonstrated that the choice of anesthetics can influence the progression of sepsis in sedated and mechanically ventilated Wistar rats that underwent CLP. Our research extended Schläpfer’s work by balancing the gender of the groups and replacing the mechanical ventilation with spontaneous breathing to reflect patient conditions in the ICU.  [1] Herrmann IK, Castellon M, Schwartz DE, Hasler M, Urner M, Hu G, Minshall RD, Beck-Schimmer B. Volatile anesthetics improve survival after cecal ligation and puncture. Anesthesiology. 2013 Oct;119(4):901-6. doi: 10.1097/ALN.0b013e3182a2a38c. PMID: 23867232; PMCID: PMC4936901.  [2] Mu J, Xie K, Hou L, Peng D, Shang L, Ji G, Li J, Lu Y, Xiong L. Subanesthetic dose of isoflurane protects against zymosan-induced generalized inflammation and its associated acute lung injury in mice. Shock. 2010 Aug;34(2):183-9. doi: 10.1097/SHK.0b013e3181cffc3f. PMID: 20160672.  [3] Lee HT, Emala CW, Joo JD, Kim M. Isoflurane improves survival and protects against renal and hepatic injury in murine septic peritonitis. Shock. 2007 Apr;27(4):373-9. doi: 10.1097/01.shk.0000248595.17130.24. PMID: 17414419.  [4] Koutsogiannaki S, Zha H, Yuki K. Volatile Anesthetic Isoflurane Attenuates Liver Injury in Experimental Polymicrobial Sepsis Model. Transl Perioper Pain Med. 2018;5(3):63-74. doi: 10.31480/2330-4871/071. Epub 2018 May 22. PMID: 29977977; PMCID: PMC6029873.  [5] Bedirli N, Demirtas CY, Akkaya T, Salman B, Alper M, Bedirli A, Pasaoglu H. Volatile anesthetic preconditioning attenuated sepsis induced lung inflammation. J Surg Res. 2012 Nov;178(1):e17-23. doi: 10.1016/j.jss.2011.12.037. Epub 2012 Mar 22. PMID: 22475355.  [6] Kharasch ED, Coopersmith CM. Sleeping to survive?: The impact of volatile anesthetics on mortality in sepsis. Anesthesiology. 2013 Oct;119(4):755-6. doi: 10.1097/ALN.0b013e3182a2a3a4. PMID: 23867233; PMCID: PMC3823825.  [7] Flondor M, Hofstetter C, Boost KA, Betz C, Homann M, Zwissler B. Isoflurane inhalation after induction of endotoxemia in rats attenuates the systemic cytokine response. Eur Surg Res. 2008;40(1):1-6. doi: 10.1159/000107614. Epub 2007 Aug 23. PMID: 17717418.  [8] Boost KA, Hofstetter C, Flondor M, Betz C, Homann M, Pfeilschifter J, Muehl H, Zwissler B. Desflurane differentially affects the release of proinflammatory cytokines in plasma and bronchoalveolar fluid of endotoxemic rats. Int J Mol Med. 2006 Jun;17(6):1139-44. PMID: 16685427.  [9] Zhang E, Zhao X, Ma H, Luo D, Hu Y, Hou L, Luo Z. A subanesthetic dose of sevoflurane combined with oxygen exerts bactericidal effects and prevents lung injury through the nitric oxide pathway during sepsis. Biomed Pharmacother. 2020 Jul;127:110169. doi: 10.1016/j.biopha.2020.110169. Epub 2020 May 8. PMID: 32403045.  [10] Schläpfer M, Piegeler T, Dull RO, Schwartz DE, Mao M, Bonini MG, Z'Graggen BR, Beck-Schimmer B, Minshall RD. Propofol increases morbidity and mortality in a rat model of sepsis. Crit Care. 2015 Feb 19;19(1):45. doi: 10.1186/s13054-015-0751-x. PMID: 25887642; PMCID: PMC4344774.  [11] Beck-Schimmer B, Baumann L, Restin T, Eugster P, Hasler M, Booy C, Schläpfer M. Sevoflurane attenuates systemic inflammation compared with propofol, but does not modulate neuro-inflammation: A laboratory rat study. Eur J Anaesthesiol. 2017 Nov;34(11):764-775. doi: 10.1097/EJA.0000000000000668. PMID: 28759530.  [12] Rudd KE, Johnson SC, Agesa KM, Shackelford KA, Tsoi D, Kievlan DR, Colombara DV, Ikuta KS, Kissoon N, Finfer S, Fleischmann-Struzek C, Machado FR, Reinhart KK, Rowan K, Seymour CW, Watson RS, West TE, Marinho F, Hay SI, Lozano R, Lopez AD, Angus DC, Murray CJL, Naghavi M. Global, regional, and national sepsis incidence and mortality, 1990-2017: analysis for the Global Burden of Disease Study. Lancet. 2020 Jan 18;395(10219):200-211. doi: 10.1016/S0140-6736(19)32989-7. PMID: 31954465; PMCID: PMC6970225.  [13] Paoli CJ, Reynolds MA, Sinha M, Gitlin M, Crouser E. Epidemiology and Costs of Sepsis in the United States-An Analysis Based on Timing of Diagnosis and Severity Level. Crit Care Med. 2018 Dec;46(12):1889-1897. doi: 10.1097/CCM.0000000000003342. PMID: 30048332; PMCID: PMC6250243. |
| 12b | Explain how the animal species and model used address the scientific objectives and, where appropriate, the relevance to human biology. | See 8a |
| 13 | **Objectives** | |
|  | Clearly describe the research question, research objectives and, where appropriate, specific hypotheses being tested. | We hypothesized that if 24 hours with volatile anesthetic agents offered beneficial immunomodulatory effects during the course of rats after CLP surgery, a 72 hour with spontaneously breathing rats may also benefit from immunomodulatory effects of volatile anesthetics. We also tested the hypothesis that the choice of anesthetics may influence the outcome of post-operative sepsis survivability. |
| 14 | **Ethical Statement** | |
|  | Provide the name of the ethical review committee or equivalent that has approved the use of animals in this study and any relevant license or protocol numbers (if applicable). If ethical approval was not sought or granted, provide a justification. | This study was approved by the University of Virginia Institutional Animal Care and Use Committee (IACUC). |
| 15 | **Housing and Husbandry** | |
|  | Provide details of housing and husbandry conditions, including any environmental enrichment. | Pathogen free Sprague Dawley rats obtained from Envigo (Indianapolis, IN, USA) were housed in standard cages (Allentown, Allentown NJ, USA) with food and water ad libitum until the time of the experiment. The animals were kept in the University of Virginia Old Medical School vivarium, with regular 12 hour light cycle, food and water ad libitum, and a toy. The animals were kept in the vivarium for minimum of 48 hours to acclimate to the new surroundings before participating in the study, in pairs. |
| 16 | **Animal care and monitoring** | |
| 16a | Describe any interventions or steps taken in the experimental protocols to reduce pain, suffering, and distress. | See 9a |
| 16b | Report any expected or unexpected adverse events. | Equipment failure during the experiment required the exclusion and the replacement of those three. |
| 16c | Describe the humane endpoints established for the study, the signs that were monitored, and the frequency of monitoring. If the study did not set humane endpoints, state this. | Euthanasia scoring system:  The following variables will be assessed every 8-12 hours in subjects subjected to cecal ligation and puncture (CLP). A combined score of 8 or maximal scores in two or more areas (e.g. activity and weight) will trigger euthanasia.  Euthanasia Scoring Sheet  Body Weight  0 Normal  1 < 10%  2 10-19%  3 20% or more  Physical Condition  Haircoat  0 Normal  1 Rough haircoat  2 Rough coat, hair loss, ungroomed  Eyes and Nose  0 Normal  1 Eyes close or squinted (no discharge)  2 Eyes close or squinted (discharge or porphyrin staining)  Behavior  Activity  0 Normal  1 Decreased activity, locomotion after slight stimulation  2 Inactive, less alert, locomotion after moderate stimulation  3 Self-mutilation, very restless or immobile or no locomotion after moderate stimulation  Posture  0 Normal  1 Sitting in hunched up position  2 Hunched posture / head on cage floor  3 Lying prone on cage floor  Additional criteria for euthanasia (even if the total score is < 8) include:   1. Weight loss > 20% which cannot be corrected in 2 days by dietary supplementation 2. Two consecutive rectal or infrared temperatures are < 32 degree Celsius |
| 17 | **Interpretation/scientific implications** | |
| 17a | Interpret the results, taking into account the study objectives and hypotheses, current theory, and other relevant studies in the literature. | There were significant difference in survivability of rats exposed to Propofol or isoflurane, for both control and post op anesthetics group. Though the statistical significance of the 0.8% isoflurane treatment group was greater than the controls, the controls group lived longer on average. Rats exposed to isoflurane during the 30-minute CLP surgery improved survivability, which supports the findings of Hermann et al [6]. Osuru et al characterized the differences in the response to sepsis while under isoflurane and Propofol and found that isoflurane and inflammation may lead to over expression of mRNA involved in the pro-inflammatory mitogen-activated protein kinase (MAPK) pathway [12] [13]. Also, increased hypoxia-inducible factor-1a expression, despite a lack of hypoxia, increased oxidative stress in the brain, and increased serum lactate (unlike propofol) [14]. These differences between VAA and propofol may indicate that the inflammatory response induced by the VAA, in the absence of infection, is protective as it prepares the body for a future infection [15].  However, the immunomodulatory advantage of VAA comes at the cost of mitochondrial autophagy and other issues associated with disrupting the electron transport chain. [12] VAA’s have been associated with neurological dysfunction [16] [17] [18] [19]. Propofol isn’t without disadvantages either. Propofol also has been implicated in metabolic abnormalities that may have a role in the neurotoxity observed in the vulnerable immature brain. [20]  [12] Osuru HP, Paila U, Ikeda K, Zuo Z, Thiele RH. Anesthesia-Sepsis-Associated Alterations in Liver Gene Expression Profiles and Mitochondrial Oxidative Phosphorylation Complexes. Front Med (Lausanne). 2020 Dec 18;7:581082. doi: 10.3389/fmed.2020.581082. PMID: 33392215; PMCID: PMC7775734.  [13] Zhang W, Liu HT. MAPK signal pathways in the regulation of cell proliferation in mammalian cells. Cell Res. 2002 Mar;12(1):9-18. doi: 10.1038/sj.cr.7290105. PMID: 11942415.  [14] Thiele RH, Osuru HP, Paila U, Ikeda K, Zuo Z. Impact of inflammation on brain subcellular energetics in anesthetized rats. BMC Neurosci. 2019 Jul 15;20(1):34. doi: 10.1186/s12868-019-0514-8. PMID: 31307382; PMCID: PMC6631861.  [15] Nedeva C, Menassa J, Puthalakath H. Sepsis: Inflammation Is a Necessary Evil. Front Cell Dev Biol. 2019 Jun 20;7:108. doi: 10.3389/fcell.2019.00108. PMID: 31281814; PMCID: PMC6596337.  [16] Zimin PI, Woods CB, Kayser EB, Ramirez JM, Morgan PG, Sedensky MM. Isoflurane disrupts excitatory neurotransmitter dynamics via inhibition of mitochondrial complex I. Br J Anaesth. 2018 May;120(5):1019-1032. doi: 10.1016/j.bja.2018.01.036. Epub 2018 Mar 13. PMID: 29661379; PMCID: PMC6200108.  [17] Zhang Y, Dong Y, Wu X, Lu Y, Xu Z, Knapp A, Yue Y, Xu T, Xie Z. The mitochondrial pathway of anesthetic isoflurane-induced apoptosis. J Biol Chem. 2010 Feb 5;285(6):4025-4037. doi: 10.1074/jbc.M109.065664. Epub 2009 Dec 10. PMID: 20007710; PMCID: PMC2823544.  [18] Bains R, Moe MC, Larsen GA, Berg-Johnsen J, Vinje ML. Volatile anaesthetics depolarize neural mitochondria by inhibiton of the electron transport chain. Acta Anaesthesiol Scand. 2006 May;50(5):572-9. doi: 10.1111/j.1399-6576.2006.00988.x. PMID: 16643227.  [19] Zhang Y, Xie Z. Anesthetics isoflurane and desflurane differently affect mitochondrial function, learning, and memory. Ann Neurol. 2012 Oct;72(4):630. doi: 10.1002/ana.23683. PMID: 23109162.  [20] Kajimoto M, Atkinson DB, Ledee DR, Kayser EB, Morgan PG, Sedensky MM, Isern NG, Des Rosiers C, Portman MA. Propofol compared with isoflurane inhibits mitochondrial metabolism in immature swine cerebral cortex. J Cereb Blood Flow Metab. 2014 Mar;34(3):514-21. doi: 10.1038/jcbfm.2013.229. Epub 2014 Jan 8. PMID: 24398942; PMCID: PMC3948133. |
| 17b | Comment on the study limitations, including potential sources of bias, limitations of the animal model, and imprecision associated with the results. | The exterior jugular vein access button was surgically installed by the Envigo surgeons, and they were installed while anesthetized with isoflurane approximately two weeks before the date of delivery. There could have been some volatile anesthetic pre-conditioning that could have biased the results towards immunomodulation for the Propofol groups 3 and 4, which would have diluted the statistically significant difference. |
| 18 | **Generalisability/translation** | |
|  | Comment on whether, and how, the findings of this study are likely to generalise to other species or experimental conditions, including any relevance to human biology (where appropriate). | A limited sampleLog-Rank test within each group for gender differences showed possibly significant difference. Further work needed to quantify optimum dosage and explain why there might be a gender difference in the effect of VAAs.  It would also be useful to investigate how long immunomodulatory effect of VAA’s last after exposure ends.  Lee et al showed that isoflurane reduced ischemia-induced brain injury in rats in post-conditioned rats, which may be a basis for a cross-over study where the rats initially are anesthetized with Propofol during the CLP surgery, and then 2% Isoflurane for 60 minutes as done in the Lee study. [1]  [1] Lee JJ, Li L, Jung HH, Zuo Z. Postconditioning with isoflurane reduced ischemia-induced brain injury in rats. Anesthesiology. 2008 Jun;108(6):1055-62. doi: 10.1097/ALN.0b013e3181730257. PMID: 18497606; PMCID: PMC2666347. |
| 19 | **Protocol registration** | |
|  | Provide a statement indicating whether a protocol (including the research question, key design features, and analysis plan) was prepared before the study, and if and where this protocol was registered. | The study was designed and approved and registered by the University of Virginia Institutional Animal Care and Use Committee (IACUC) before we engaged in any research activities. |
|  | **Data Access** | |
|  | Provide a statement describing if and where study data are available. | The online version of this article contains supplementary material, which is available to authorized users. |
| 21 | **Declaration of interests** | |
| 21a | Declare any potential conflicts of interest, including financial and nonfinancial. If none exist, this should be stated. | None of the investigators had any conflict of interest that could have influenced the outcome of this study. |
| 21b | List all funding sources (including grant identifier) and the role of the funder(s) in the design, analysis, and reporting of the study. | This study was partially funded by the National Institute of Health. |

Full NLM Citations:

1. Herrmann IK, Castellon M, Schwartz DE, Hasler M, Urner M, Hu G, Minshall RD, Beck-Schimmer B. Volatile anesthetics improve survival after cecal ligation and puncture. Anesthesiology. 2013 Oct;119(4):901-6. doi: 10.1097/ALN.0b013e3182a2a38c. PMID: 23867232; PMCID: PMC4936901.

2. Mu J, Xie K, Hou L, Peng D, Shang L, Ji G, Li J, Lu Y, Xiong L. Subanesthetic dose of isoflurane protects against zymosan-induced generalized inflammation and its associated acute lung injury in mice. Shock. 2010 Aug;34(2):183-9. doi: 10.1097/SHK.0b013e3181cffc3f. PMID: 20160672.

3. Lee HT, Emala CW, Joo JD, Kim M. Isoflurane improves survival and protects against renal and hepatic injury in murine septic peritonitis. Shock. 2007 Apr;27(4):373-9. doi: 10.1097/01.shk.0000248595.17130.24. PMID: 17414419.

4. Koutsogiannaki S, Zha H, Yuki K. Volatile Anesthetic Isoflurane Attenuates Liver Injury in Experimental Polymicrobial Sepsis Model. Transl Perioper Pain Med. 2018;5(3):63-74. doi: 10.31480/2330-4871/071. Epub 2018 May 22. PMID: 29977977; PMCID: PMC6029873.

5. Bedirli N, Demirtas CY, Akkaya T, Salman B, Alper M, Bedirli A, Pasaoglu H. Volatile anesthetic preconditioning attenuated sepsis induced lung inflammation. J Surg Res. 2012 Nov;178(1):e17-23. doi: 10.1016/j.jss.2011.12.037. Epub 2012 Mar 22. PMID: 22475355.

6. Kharasch ED, Coopersmith CM. Sleeping to survive?: The impact of volatile anesthetics on mortality in sepsis. Anesthesiology. 2013 Oct;119(4):755-6. doi: 10.1097/ALN.0b013e3182a2a3a4. PMID: 23867233; PMCID: PMC3823825.

7. Flondor M, Hofstetter C, Boost KA, Betz C, Homann M, Zwissler B. Isoflurane inhalation after induction of endotoxemia in rats attenuates the systemic cytokine response. Eur Surg Res. 2008;40(1):1-6. doi: 10.1159/000107614. Epub 2007 Aug 23. PMID: 17717418.

8. Boost KA, Hofstetter C, Flondor M, Betz C, Homann M, Pfeilschifter J, Muehl H, Zwissler B. Desflurane differentially affects the release of proinflammatory cytokines in plasma and bronchoalveolar fluid of endotoxemic rats. Int J Mol Med. 2006 Jun;17(6):1139-44. PMID: 16685427.

9. Zhang E, Zhao X, Ma H, Luo D, Hu Y, Hou L, Luo Z. A subanesthetic dose of sevoflurane combined with oxygen exerts bactericidal effects and prevents lung injury through the nitric oxide pathway during sepsis. Biomed Pharmacother. 2020 Jul;127:110169. doi: 10.1016/j.biopha.2020.110169. Epub 2020 May 8. PMID: 32403045.

10. Schläpfer M, Piegeler T, Dull RO, Schwartz DE, Mao M, Bonini MG, Z'Graggen BR, Beck-Schimmer B, Minshall RD. Propofol increases morbidity and mortality in a rat model of sepsis. Crit Care. 2015 Feb 19;19(1):45. doi: 10.1186/s13054-015-0751-x. PMID: 25887642; PMCID: PMC4344774.

11. Beck-Schimmer B, Baumann L, Restin T, Eugster P, Hasler M, Booy C, Schläpfer M. Sevoflurane attenuates systemic inflammation compared with propofol, but does not modulate neuro-inflammation: A laboratory rat study. Eur J Anaesthesiol. 2017 Nov;34(11):764-775. doi: 10.1097/EJA.0000000000000668. PMID: 28759530.

12. Osuru HP, Paila U, Ikeda K, Zuo Z, Thiele RH. Anesthesia-Sepsis-Associated Alterations in Liver Gene Expression Profiles and Mitochondrial Oxidative Phosphorylation Complexes. Front Med (Lausanne). 2020 Dec 18;7:581082. doi: 10.3389/fmed.2020.581082. PMID: 33392215; PMCID: PMC7775734.

13. Thiele RH, Osuru HP, Paila U, Ikeda K, Zuo Z. Impact of inflammation on brain subcellular energetics in anesthetized rats. BMC Neurosci. 2019 Jul 15;20(1):34. doi: 10.1186/s12868-019-0514-8. PMID: 31307382; PMCID: PMC6631861.

14. Nedeva C, Menassa J, Puthalakath H. Sepsis: Inflammation Is a Necessary Evil. Front Cell Dev Biol. 2019 Jun 20;7:108. doi: 10.3389/fcell.2019.00108. PMID: 31281814; PMCID: PMC6596337.

Author’s Notes:

Items 1-13 of 13 ([Display the 13 citations in PubMed](https://pubmed.ncbi.nlm.nih.gov/?term=20160672,28759530,17414419,16269307,16685427,17717418,29977977,23867233,32403045,23867232,25887642,31307382,22475355))

| 1. | [Subanesthetic dose of isoflurane protects against zymosan-induced generalized inflammation and its associated acute lung injury in mice.](https://pubmed.ncbi.nlm.nih.gov/20160672/)  Mu J, Xie K, Hou L, Peng D, Shang L, Ji G, Li J, Lu Y, Xiong L.  Shock. 2010 Aug;34(2):183-9. doi: 10.1097/SHK.0b013e3181cffc3f.  PMID: 20160672 |
| --- | --- |
| 2. | [Sevoflurane attenuates systemic inflammation compared with propofol, but does not modulate neuro-inflammation: A laboratory rat study.](https://pubmed.ncbi.nlm.nih.gov/28759530/)  Beck-Schimmer B, Baumann L, Restin T, Eugster P, Hasler M, Booy C, Schläpfer M.  Eur J Anaesthesiol. 2017 Nov;34(11):764-775. doi: 10.1097/EJA.0000000000000668.  PMID: 28759530 |
| 3. | [Isoflurane improves survival and protects against renal and hepatic injury in murine septic peritonitis.](https://pubmed.ncbi.nlm.nih.gov/17414419/)  Lee HT, Emala CW, Joo JD, Kim M.  Shock. 2007 Apr;27(4):373-9. doi: 10.1097/01.shk.0000248595.17130.24.  PMID: 17414419 |
| 4. | [Anesthesia-specific protection from endotoxic shock is not mediated through the vagus nerve.](https://pubmed.ncbi.nlm.nih.gov/16269307/)  Fuentes JM, Hanly EJ, Aurora AR, De Maio A, Talamini MA.  Surgery. 2005 Oct;138(4):766-71. doi: 10.1016/j.surg.2005.06.057.  PMID: 16269307 |
| 5. | [Desflurane differentially affects the release of proinflammatory cytokines in plasma and bronchoalveolar fluid of endotoxemic rats.](https://pubmed.ncbi.nlm.nih.gov/16685427/)  Boost KA, Hofstetter C, Flondor M, Betz C, Homann M, Pfeilschifter J, Muehl H, Zwissler B.  Int J Mol Med. 2006 Jun;17(6):1139-44.  PMID: 16685427 |
| 6. | [Isoflurane inhalation after induction of endotoxemia in rats attenuates the systemic cytokine response.](https://pubmed.ncbi.nlm.nih.gov/17717418/)  Flondor M, Hofstetter C, Boost KA, Betz C, Homann M, Zwissler B.  Eur Surg Res. 2008;40(1):1-6. doi: 10.1159/000107614. Epub 2007 Aug 23.  PMID: 17717418 |
| 7. | [Volatile Anesthetic Isoflurane Attenuates Liver Injury in Experimental Polymicrobial Sepsis Model.](https://pubmed.ncbi.nlm.nih.gov/29977977/)  Koutsogiannaki S, Zha H, Yuki K.  Transl Perioper Pain Med. 2018;5(3):63-74. doi: 10.31480/2330-4871/071. Epub 2018 May 22.  PMID: 29977977 Free PMC article. |
| 8. | [Sleeping to survive?: The impact of volatile anesthetics on mortality in sepsis.](https://pubmed.ncbi.nlm.nih.gov/23867233/)  Kharasch ED, Coopersmith CM.  Anesthesiology. 2013 Oct;119(4):755-6. doi: 10.1097/ALN.0b013e3182a2a3a4.  PMID: 23867233 Free PMC article. No abstract available. |
| 9. | [A subanesthetic dose of sevoflurane combined with oxygen exerts bactericidal effects and prevents lung injury through the nitric oxide pathway during sepsis.](https://pubmed.ncbi.nlm.nih.gov/32403045/)  Zhang E, Zhao X, Ma H, Luo D, Hu Y, Hou L, Luo Z.  Biomed Pharmacother. 2020 Jul;127:110169. doi: 10.1016/j.biopha.2020.110169. Epub 2020 May 8.  PMID: 32403045 |
| 10. | [Volatile anesthetics improve survival after cecal ligation and puncture.](https://pubmed.ncbi.nlm.nih.gov/23867232/)  Herrmann IK, Castellon M, Schwartz DE, Hasler M, Urner M, Hu G, Minshall RD, Beck-Schimmer B.  Anesthesiology. 2013 Oct;119(4):901-6. doi: 10.1097/ALN.0b013e3182a2a38c.  PMID: 23867232 Free PMC article. |
| 11. | [Propofol increases morbidity and mortality in a rat model of sepsis.](https://pubmed.ncbi.nlm.nih.gov/25887642/)  Schläpfer M, Piegeler T, Dull RO, Schwartz DE, Mao M, Bonini MG, Z'Graggen BR, Beck-Schimmer B, Minshall RD.  Crit Care. 2015 Feb 19;19(1):45. doi: 10.1186/s13054-015-0751-x.  PMID: 25887642 Free PMC article. |
| 12. | [Impact of inflammation on brain subcellular energetics in anesthetized rats.](https://pubmed.ncbi.nlm.nih.gov/31307382/)  Thiele RH, Osuru HP, Paila U, Ikeda K, Zuo Z.  BMC Neurosci. 2019 Jul 15;20(1):34. doi: 10.1186/s12868-019-0514-8.  PMID: 31307382 Free PMC article. |
| 13. | [Volatile anesthetic preconditioning attenuated sepsis induced lung inflammation.](https://pubmed.ncbi.nlm.nih.gov/22475355/)  Bedirli N, Demirtas CY, Akkaya T, Salman B, Alper M, Bedirli A, Pasaoglu H.  J Surg Res. 2012 Nov;178(1):e17-23. doi: 10.1016/j.jss.2011.12.037. Epub 2012 Mar 22.  PMID: 22475355 |

Items 1-13 of 13 ([Display the 13 citations in PubMed](https://pubmed.ncbi.nlm.nih.gov/?term=26854136,29661379,30989480,31545457,16249673,22368036,20007710,16643227,18212570,19568162,23109162,24398942,10969309))

| 1. | [Exposure of isoflurane-treated cells to hyperoxia decreases cell viability and activates the mitochondrial apoptotic pathway.](https://pubmed.ncbi.nlm.nih.gov/26854136/)  Kim GH, Lee JJ, Lee SH, Chung YH, Cho HS, Kim JA, Kim MK.  Brain Res. 2016 Apr 1;1636:13-20. doi: 10.1016/j.brainres.2016.01.052. Epub 2016 Feb 5.  PMID: 26854136 |
| --- | --- |
| 2. | [Isoflurane disrupts excitatory neurotransmitter dynamics via inhibition of mitochondrial complex I.](https://pubmed.ncbi.nlm.nih.gov/29661379/)  Zimin PI, Woods CB, Kayser EB, Ramirez JM, Morgan PG, Sedensky MM.  Br J Anaesth. 2018 May;120(5):1019-1032. doi: 10.1016/j.bja.2018.01.036. Epub 2018 Mar 13.  PMID: 29661379 Free PMC article. |
| 3. | [Lidocaine Attenuates Cognitive Impairment After Isoflurane Anesthesia by Reducing Mitochondrial Damage.](https://pubmed.ncbi.nlm.nih.gov/30989480/)  Li J, Zhu X, Yang S, Xu H, Guo M, Yao Y, Huang Z, Lin D.  Neurochem Res. 2019 Jul;44(7):1703-1714. doi: 10.1007/s11064-019-02799-0. Epub 2019 Apr 15.  PMID: 30989480 |
| 4. | [Effects of isoflurane on complex II‑associated mitochondrial respiration and reactive oxygen species production: Roles of nitric oxide and mitochondrial KATP channels.](https://pubmed.ncbi.nlm.nih.gov/31545457/)  Wang J, Sun J, Qiao S, Li H, Che T, Wang C, An J.  Mol Med Rep. 2019 Nov;20(5):4383-4390. doi: 10.3892/mmr.2019.10658. Epub 2019 Sep 9.  PMID: 31545457 |
| 5. | [Isoflurane postconditioning prevents opening of the mitochondrial permeability transition pore through inhibition of glycogen synthase kinase 3beta.](https://pubmed.ncbi.nlm.nih.gov/16249673/)  Feng J, Lucchinetti E, Ahuja P, Pasch T, Perriard JC, Zaugg M.  Anesthesiology. 2005 Nov;103(5):987-95. doi: 10.1097/00000542-200511000-00013.  PMID: 16249673 |
| 6. | [Anesthetics isoflurane and desflurane differently affect mitochondrial function, learning, and memory.](https://pubmed.ncbi.nlm.nih.gov/22368036/)  Zhang Y, Xu Z, Wang H, Dong Y, Shi HN, Culley DJ, Crosby G, Marcantonio ER, Tanzi RE, Xie Z.  Ann Neurol. 2012 May;71(5):687-98. doi: 10.1002/ana.23536. Epub 2012 Feb 24.  PMID: 22368036 Free PMC article. |
| 7. | [The mitochondrial pathway of anesthetic isoflurane-induced apoptosis.](https://pubmed.ncbi.nlm.nih.gov/20007710/)  Zhang Y, Dong Y, Wu X, Lu Y, Xu Z, Knapp A, Yue Y, Xu T, Xie Z.  J Biol Chem. 2010 Feb 5;285(6):4025-37. doi: 10.1074/jbc.M109.065664. Epub 2009 Dec 10.  PMID: 20007710 Free PMC article. |
| 8. | [Volatile anaesthetics depolarize neural mitochondria by inhibiton of the electron transport chain.](https://pubmed.ncbi.nlm.nih.gov/16643227/)  Bains R, Moe MC, Larsen GA, Berg-Johnsen J, Vinje ML.  Acta Anaesthesiol Scand. 2006 May;50(5):572-9. doi: 10.1111/j.1399-6576.2006.00988.x.  PMID: 16643227 |
| 9. | [The common inhalational anesthetic isoflurane induces apoptosis via activation of inositol 1,4,5-trisphosphate receptors.](https://pubmed.ncbi.nlm.nih.gov/18212570/)  Wei H, Liang G, Yang H, Wang Q, Hawkins B, Madesh M, Wang S, Eckenhoff RG.  Anesthesiology. 2008 Feb;108(2):251-60. doi: 10.1097/01.anes.0000299435.59242.0e.  PMID: 18212570 |
| 10. | [Anesthetic-induced preconditioning delays opening of mitochondrial permeability transition pore via protein Kinase C-epsilon-mediated pathway.](https://pubmed.ncbi.nlm.nih.gov/19568162/)  Pravdic D, Sedlic F, Mio Y, Vladic N, Bienengraeber M, Bosnjak ZJ.  Anesthesiology. 2009 Aug;111(2):267-74. doi: 10.1097/ALN.0b013e3181a91957.  PMID: 19568162 Free PMC article. |
| 11. | [Anesthetics isoflurane and desflurane differently affect mitochondrial function, learning, and memory.](https://pubmed.ncbi.nlm.nih.gov/23109162/)  Zhang Y, Xie Z.  Ann Neurol. 2012 Oct;72(4):630. doi: 10.1002/ana.23683.  PMID: 23109162 No abstract available. |
| 12. | [Propofol compared with isoflurane inhibits mitochondrial metabolism in immature swine cerebral cortex.](https://pubmed.ncbi.nlm.nih.gov/24398942/)  Kajimoto M, Atkinson DB, Ledee DR, Kayser EB, Morgan PG, Sedensky MM, Isern NG, Des Rosiers C, Portman MA.  J Cereb Blood Flow Metab. 2014 Mar;34(3):514-21. doi: 10.1038/jcbfm.2013.229. Epub 2014 Jan 8.  PMID: 24398942 Free PMC article. |
| 13. | [Prevention of isoflurane-induced preconditioning by 5-hydroxydecanoate and gadolinium: possible involvement of mitochondrial adenosine triphosphate-sensitive potassium and stretch-activated channels.](https://pubmed.ncbi.nlm.nih.gov/10969309/)  Piriou V, Chiari P, Knezynski S, Bastien O, Loufoua J, Lehot JJ, Foëx P, Annat G, Ovize M.  Anesthesiology. 2000 Sep;93(3):756-64. doi: 10.1097/00000542-200009000-00025.  PMID: 10969309 |
