# Supplementary material for: Intraoperative administration of isoflurane improves survival in rats exposed to caecal ligation and puncture
Source: BJA Open. 2022 May 21;2:100014. doi: 10.1016/j.bjao.2022.100014 (PMC10430831; doi:10.1016/j.bjao.2022.100014)
Supplement: Multimedia component 2 [file mmc3.docx]

| Group | Gender |  | Mass before CLP (g) | Mass at death (g) | Change in Mass |
| --- | --- | --- | --- | --- | --- |
| Long | F | Mean | 267.33 | 265.56 | -1.78 |
| Isoflurane | | Median | 266.00 | 262.00 | -2.00 |
| Group |  | SD | 22.19 | 21.76 | 1.93 |
| Short | F | Mean | 263.33 | 252.33 | -11.00 |
| Isoflurane | | Median | 261.00 | 250.00 | -5.00 |
| Group |  | SD | 14.02 | 15.08 | 10.17 |
| Long | F | Mean | 261.89 | 256.67 | -5.22 |
| Propofol |  | Median | 256.00 | 250.00 | -2.00 |
| Group |  | SD | 13.63 | 13.89 | 9.51 |
| Short | F | Mean | 265.22 | 261.78 | -3.44 |
| Propofol |  | Median | 265.00 | 257.00 | -3.00 |
| Group |  | SD | 14.32 | 13.98 | 2.17 |
| Long | M | Mean | 307.44 | 305.44 | -2.00 |
| Isoflurane | | Median | 294.00 | 290.00 | 0.00 |
| Group |  | SD | 27.02 | 28.30 | 3.89 |
| Short | M | Mean | 299.78 | 287.22 | -12.56 |
| Isoflurane | | Median | 307.00 | 290.00 | -6.00 |
| Group |  | SD | 15.54 | 19.43 | 15.79 |
| Long | M | Mean | 309.11 | 308.44 | -0.67 |
| Propofol |  | Median | 296.00 | 296.00 | 0.00 |
| Group |  | SD | 31.57 | 34.11 | 4.42 |
| Short | M | Mean | 298.33 | 294.56 | -3.78 |
| Propofol |  | Median | 298.00 | 281.00 | -3.00 |
| Group |  | SD | 16.31 | 20.73 | 7.63 |

Table 3. Mass of rats within each group, according to sex.

| Comparison of Survival Curves | Male and Female |  | Females Only |  | Males Only |  |
| --- | --- | --- | --- | --- | --- | --- |
|  |  |  |  |  |  |  |
| Log-rank (Mantel-Cox) test |  |  |  |  |  |  |
| Chi square | 15.44 |  | 5.217 |  | 11.96 |  |
| df | 1 |  | 1 |  | 1 |  |
| P value | <0.0001 |  | 0.0224 |  | 0.0005 |  |
| P value summary | **** |  | * |  | *** |  |
| Are the survival curves sig different? | Yes |  | Yes |  | Yes |  |
| Median survival |  |  |  |  |  |  |
| Long Isoflurane | 1.425 |  | 1.425 |  | 1.425 |  |
| Long Propofol | 0.8163 |  | 0.7653 |  | 1.006 |  |
| Ratio (and its reciprocal) | 1.746 | 0.5729 | 1.862 | 0.537 | 1.416 | 0.7061 |
| 95% CI of ratio | [0.9083, 3.355] | [0.2981, 1.101] | [0.7391, 4.691] | [0.2132, 1.353] | [0.5621, 3.568] | [0.2803, 1.779] |
|  |  |  |  |  |  |  |
| Hazard Ratio (Mantel-Haenszel) | A/B | B/A | A/B | B/A | A/B | B/A |
| Ratio (and its reciprocal) | 0.1898 | 5.269 | 0.2708 | 3.693 | 0.107 | 9.346 |
| 95% CI of ratio | [0.08284, 0.4348] | [2.300, 12.07] | [0.08826, 0.8308] | [1.204, 11.33] | [0.03014, 0.3798] | [2.633, 33.18] |
|  |  |  |  |  |  |  |
| Hazard Ratio (logrank) | A/B | B/A | A/B | B/A | A/B | B/A |
| Ratio (and its reciprocal) | 0.3192 | 3.133 | 0.3853 | 2.595 | 0.2544 | 3.931 |
| 95% CI of ratio | [0.1489, 0.6844] | [1.461, 6.717] | [0.1374, 1.080] | [0.9256, 7.277] | [0.08063, 0.8026] | [1.246, 12.40] |

Table 4. Log-Rank and Hazard Ratio calculated from groups LI and LP from Kaplan-Meyer survival comparisons data.

| Comparison of Survival Curves | Male and Female |  | Females Only |  | Males Only |  |
| --- | --- | --- | --- | --- | --- | --- |
|  |  |  |  |  |  |  |
| Log-rank (Mantel-Cox) test |  |  |  |  |  |  |
| Chi square | 7.553 |  | 7.784 |  | 1.269 |  |
| df | 1 |  | 1 |  | 1 |  |
| P value | 0.006 |  | 0.0053 |  | 0.2599 |  |
| P value summary | ** |  | ** |  | ns |  |
| Are the survival curves sig different? | Yes |  | Yes |  | No |  |
| Median survival |  |  |  |  |  |  |
| Short Isoflurane | 1.372 |  | 1.408 |  | 1.335 |  |
| Short Propofol | 0.9872 |  | 0.7813 |  | 1.099 |  |
| Ratio (and its reciprocal) | 1.389 | 0.7197 | 1.802 | 0.555 | 1.215 | 0.8232 |
| 95% CI of ratio | [0.7229, 2.670] | [0.3745, 1.383] | [0.7152, 4.539] | [0.2203, 1.398] | [0.4822, 3.060] | [0.3268, 2.074] |
|  |  |  |  |  |  |  |
| Hazard Ratio (Mantel-Haenszel) | A/B | B/A | A/B | B/A | A/B | B/A |
| Ratio (and its reciprocal) | 0.3492 | 2.864 | 0.1866 | 5.358 | 0.562 | 1.779 |
| 95% CI of ratio | [0.1649, 0.7395] | [1.352, 6.066] | [0.05739, 0.6069] | [1.648, 17.42] | [0.2062, 1.532] | [0.6529, 4.849] |
|  |  |  |  |  |  |  |
| Hazard Ratio (logrank) | A/B | B/A | A/B | B/A | A/B | B/A |
| Ratio (and its reciprocal) | 0.4298 | 2.327 | 0.3199 | 3.126 | 0.6068 | 1.648 |
| 95% CI of ratio | [0.2108, 0.8764] | [1.141, 4.744] | [0.1089, 0.9402] | [1.064, 9.187] | [0.2340, 1.574] | [0.6355, 4.274] |

Table 5. Log-Rank and Hazard Ratio calculated from group SI and SP from Kaplan-Meyer survival comparisons data.

| Comparison of Survival Curves | Males and Females |  | Females Only |  | Males Only |  |
| --- | --- | --- | --- | --- | --- | --- |
|  |  |  |  |  |  |  |
| Log-rank (Mantel-Cox) test |  |  |  |  |  |  |
| Chi square | 0.218 |  | 0.3704 |  | 0.00001991 |  |
| df | 1 |  | 1 |  | 1 |  |
| P value | 0.6406 |  | 0.5428 |  | 0.9964 |  |
| P value summary | ns |  | ns |  | ns |  |
| Are the survival curves sig different? | No |  | No |  | No |  |
| Median survival |  |  |  |  |  |  |
| Long Isoflurane | 1.425 |  | 1.425 |  | 1.425 |  |
| Short Isoflurane | 1.372 |  | 1.408 |  | 1.335 |  |
| Ratio (and its reciprocal) | 1.039 | 0.9625 | 1.012 | 0.9878 | 1.067 | 0.9371 |
| 95% CI of ratio | [0.5406, 1.997] | [0.5008, 1.850] | [0.4018, 2.550] | [0.3921, 2.489] | [0.4236, 2.688] | [0.3720, 2.361] |
|  |  |  |  |  |  |  |
| Hazard Ratio (Mantel-Haenszel) | A/B | B/A | A/B | B/A | A/B | B/A |
| Ratio (and its reciprocal) | 1.182 | 0.8457 | 1.358 | 0.7362 | 0.9977 | 1.002 |
| 95% CI of ratio | [0.5852, 2.389] | [0.4186, 1.709] | [0.5066, 3.642] | [0.2745, 1.974] | [0.3668, 2.714] | [0.3685, 2.726] |
|  |  |  |  |  |  |  |
| Hazard Ratio (logrank) | A/B | B/A | A/B | B/A | A/B | B/A |
| Ratio (and its reciprocal) | 1.156 | 0.8652 | 1.31 | 0.7631 | 0.9981 | 1.002 |
| 95% CI of ratio | [0.6004, 2.225] | [0.4494, 1.666] | [0.5158, 3.329] | [0.3004, 1.939] | [0.3962, 2.514] | [0.3977, 2.524] |

Table 6. Log-Rank and Hazard Ratio calculated with groups LI and SI from Kaplan-Meyer survival comparisons data a.

| Comparison of Survival Curves |  |  | Females Only |  | Males Only |  |
| --- | --- | --- | --- | --- | --- | --- |
|  |  |  |  |  |  |  |
| Log-rank (Mantel-Cox) test |  |  |  |  |  |  |
| Chi square | 0.2302 |  | 0.2278 |  | 1.201 |  |
| df | 1 |  | 1 |  | 1 |  |
| P value | 0.6314 |  | 0.6331 |  | 0.2731 |  |
| P value summary | ns |  | ns |  | ns |  |
| Are the survival curves sig different? | No |  | No |  | No |  |
| Median survival |  |  |  |  |  |  |
| Long Propofol | 0.8163 |  | 0.7653 |  | 1.006 |  |
| Short Propofol | 0.9872 |  | 0.7813 |  | 1.099 |  |
| Ratio (and its reciprocal) | 0.8269 | 1.209 | 0.9796 | 1.021 | 0.9154 | 1.092 |
| 95% CI of ratio | [0.4303, 1.589] | [0.6292, 2.324] | [0.3888, 2.468] | [0.4052, 2.572] | [0.3633, 2.306] | [0.4337, 2.752] |
|  |  |  |  |  |  |  |
| Hazard Ratio (Mantel-Haenszel) | A/B | B/A | A/B | B/A | A/B | B/A |
| Ratio (and its reciprocal) | 1.178 | 0.8488 | 0.7844 | 1.275 | 1.749 | 0.5716 |
| 95% CI of ratio | [0.6029, 2.302] | [0.4344, 1.659] | [0.2893, 2.127] | [0.4702, 3.457] | [0.6434, 4.756] | [0.2103, 1.554] |
|  |  |  |  |  |  |  |
| Hazard Ratio (logrank) | A/B | B/A | A/B | B/A | A/B | B/A |
| Ratio (and its reciprocal) | 1.169 | 0.8553 | 0.8112 | 1.233 | 1.627 | 0.6147 |
| 95% CI of ratio | [0.6071, 2.252] | [0.4441, 1.647] | [0.3204, 2.054] | [0.4868, 3.121] | [0.6283, 4.213] | [0.2374, 1.592] |

Table 7. Log-Rank and Hazard Ratio calculated with groups LP and SP from Kaplan-Meyer survival comparisons data.
